# Analysing Twitter and web queries for flu trend prediction

**DOI:** 10.1186/1742-4682-11-S1-S6

**Published:** 2014-05-07

**Authors:** José Carlos Santos, Sérgio Matos

**Affiliations:** 1 DETI/IEETA, Universidade de Aveiro, Aveiro, 3810-193, Portugal

## Abstract

**Background:**

Social media platforms encourage people to share diverse aspects of their daily life. Among these, shared health related information might be used to infer health status and incidence rates for specific conditions or symptoms. In this work, we present an infodemiology study that evaluates the use of Twitter messages and search engine query logs to estimate and predict the incidence rate of influenza like illness in Portugal.

**Results:**

Based on a manually classified dataset of 2704 tweets from Portugal, we selected a set of 650 textual features to train a Naïve Bayes classifier to identify tweets mentioning flu or flu-like illness or symptoms. We obtained a precision of 0.78 and an F-measure of 0.83, based on cross validation over the complete annotated set. Furthermore, we trained a multiple linear regression model to estimate the health-monitoring data from the Influenzanet project, using as predictors the relative frequencies obtained from the tweet classification results and from query logs, and achieved a correlation ratio of 0.89 (*p *< 0.001). These classification and regression models were also applied to estimate the flu incidence in the following flu season, achieving a correlation of 0.72.

**Conclusions:**

Previous studies addressing the estimation of disease incidence based on user-generated content have mostly focused on the english language. Our results further validate those studies and show that by changing the initial steps of data preprocessing and feature extraction and selection, the proposed approaches can be adapted to other languages. Additionally, we investigated whether the predictive model created can be applied to data from the subsequent flu season. In this case, although the prediction result was good, an initial phase to adapt the regression model could be necessary to achieve more robust results.

## Background

With the establishment of the Web2.0 paradigm, the Internet became a means of disseminating personal information rather than being used only as a source of information. Also thanks to the widespread access to computers, and to the appearance of other more mobile platforms such as smartphones and tablets, large quantities of user generated content (UGC) are currently created every day, on web pages, blogs and through social networking services like Twitter or Facebook. This content includes for example, personal experiences, knowledge, product reviews, and health information [1,2], representing great opportunities with many possible applications. Mining these data provides an instantaneous snapshot of the public's opinions, and longitudinal tracking allows identification of changes in opinions [3]. Twitter [4], for example, offers a micro-blogging service that allows users to communicate through status updates limited to 140 characters, commonly referred to as "tweets". It has over 200 million active users, and around 400 million tweets are published daily.

Among the various forms of content that is created, people often use social networking services to share personal health related information, such as the appearance of flu symptoms or the recovery of those symptoms. Other types of user-generated content, such as Internet searches or comments to news articles, may also contain information related to some of these aspects. Thus, this information can be used to identify disease cases and estimate the disease incidence rate through time. The use of these different forms of internet data to infer and predict disease incidence is the subject of infodemiology, an emerging field of study also focused on syndromic surveillance and on measuring and tracking the diffusion of health information over the internet [5].

Various previous works have used Twitter and other user-generated data to assess and categorize the kind of information sought by individuals, to infer health status or measure the spread of a disease in a population [6]. In Lyon et al. [7], for example, the authors compared three web-based biosecurity intelligence systems and highlighted the value of social media, namely Twitter, in terms of the speed the information is passed and also because many issues or messages were not disseminated through other means. Chew and Eysenbach [3] suggested a complementary infoveillance approach during the 2009 H1N1 pandemic, using Twitter. They applied content and sentiment analysis to 2 million tweets containing the keywords "swine flu", "swineflu", or "H1N1". For this, they created a range of queries related to different content categories, and showed that the results of these queries correlated well with the results of manual coding, suggesting that near real-time content and sentiment analysis could be achieved, allowing monitoring large amounts of textual data over time. Signorini et al. [8] collected tweets matching a set of 15 pre-specified search terms including "flu", "vaccine", "tamiflu", and "h1n1" and applied content analysis and regression models to measure and monitor public concern and levels of disease during the H1N1 pandemic in the United States. Using a regression model trained on 1 million influenza-related tweets and using the incidence rates reported by the Centers for Disease Control (CDC) as reference, the authors reported errors ranging from 0.04% to 0.93% for the estimation of influenza-like illness levels. Chunara et al. [9] analysed cholera-related tweets published during the first 100 days of the 2010 Haitian cholera outbreak. For this, all tweets published in this period and containing the word "cholera" or the hashtag "#cholera" were considered, and these data were compared to data from two sources: HealthMap, an automated surveillance platform, and the Haitian Ministry of Public Health (MSPP). They showed good correlation between Twitter and HealthMap data, and showed a good correlation (0.83) between Twitter and MSPP data in the initial period of the outbreak, although this value decreased to 0.25 when the complete 100 days period was considered.

Aramaki et al. [10] applied SVM machine learning techniques to Twitter messages to predict influenza rates in Japan. Lampos and Cristianini [11] and Culotta [12,13] analysed Twitter messages using regression models, in the United Kingdom and the United States respectively, obtaining correlation rates of approximately 0.95. More recently, Lee et al. [14] proposed a tool for real-time disease surveillance using Twitter data, showing daily activity of the disease and corresponding symptoms automatically mined from the text, as well as geographical incidence.

Different works also rely on query logs to track influenza activity. One of earliest works in this field was performed by Eysenbach [15], who observed a Pearson's correlation coefficient of 0.91 between clicks on a keyword-triggered advert in the Google search engine with epidemiological data from Canada. This work was later extended by Ginsberg et al. [16] who presented Google Flu Trends, a tool based on Google search queries and that achieved an average correlation of 0.97 when compared against the ILI percentages provided by the Centers for Disease Control (CDC).

The greatest advantage of these methods over traditional ones is instant feedback: while health reports are published in a weekly or monthly basis, Twitter and/or query log analyses can be obtained almost instantly. This characteristic is of extreme importance because early stage detection can reduce the impact of epidemic breakouts [10,16]. In this work, we adapted previously described methods in order to estimate the occurrence rate of influenza in Portugal using user-generated content from different sources, namely tweets and query logs, combined through multiple linear regression models. We take a two-step approach, as used in similar studies: first, tweets and user queries are selected according to manually crafted regular expressions, and tweets are further classified as 'positive' or 'negative', according to whether the text points to the occurrence of flu or not; secondly, we use the relative frequencies of the selected tweets and search queries to estimate flu incidence rates as predicted through an online self-reporting scheme. We show validation results for each of these steps and also evaluate if the trained models can be directly applied in the following flu seasons, allowing to track disease trends.

The article is organized as follows: the next section describes the methodology, followed by a presentation and discussion of the obtained results; finally, some conclusions and proposals for further studies are presented in the last section.

## Methods

The main objective of this work was to evaluate if user-generated content could be used to create a reliable predictive model to obtain instant feedback regarding the incidence of flu in Portugal, allowing to track its changes over time during the main flu period. In order to assess the performance of our approach, we compare it to epidemiological results from Influenzanet [17], a health-monitoring project related to flu. Influenzanet data is collected from several participants who sign up to the project and report any influenza symptoms, such as fevers or headaches, on a weekly basis. Influenzanet results are presented as weekly activity levels, defined as the number of onsets of the symptoms on a given week divided by the number of participant-weeks. As of May 2013, over 41200 volunteers from nine european countries were contributing to the Influenzanet project. In Portugal, 1552 participants were registered and contributing to the data at this time.

Our proposed method is based on the analysis of tweets and web search queries. We start by identifying tweets and queries that are relevant for indicating the presence of flu or flu symptoms. This might be a statement on a Twitter status or a web search for flu medication, for example. We then calculate the weekly relative frequencies of flu related tweets and web queries, that is, the fraction of tweets (queries) in a given week that are related to flu. Given these values we trained various regression models using the Influenzanet results as the dependent variable, or predictand. The data were time aligned with the Influenzanet results. We used training data from nearly 14 million tweets originating in Portugal and covering the period between March 2011 and February 2012, an average of 324 thousand tweets per week. An initial analysis showed that a large quantity of tweets including links to web addresses (URLs) were from news sources, or were linking to news stories. Therefore, all tweets containing an URL were removed from the dataset in order to avoid bias. Similarly, replies (re-tweets) were also excluded. Besides Twitter, we also used around 15 million query log entries from the SAPO [18] search platform from December 2011 to May 2012, an average of 780 thousand log entries per week.

In order to verify the possible application of the proposed method in a real scenario, we tested the hypothesis that the classification and regression models trained in one flu season could be applied in the following season. This would allow measuring the incidence of flu on a weekly basis, in almost real time. For this, we obtained a total of 24 million tweets, created from December 2012 to April 2013, an average of 1,1 million tweets per week. From the web search logs, we obtained a total of 14 million queries for the same period, an average of 650 thousand searches per week.

### Regular expressions

We start by applying regular expressions in order to capture tweets and queries that contain influenza related words. For the queries, we used a simple regular expression that matches "*gripe*" (the Portuguese word for influenza) word derivations: "*(en)?grip[a-z]+*". A set of 1547 searches was identified, an average of 47 searches per week.

For filtering the Twitter data we used a more complex expression, since tweets may contain a more descriptive account of someone's health status. The regular expression was built according to common insights about how people describe flu and flu-like symptoms, and can be divided into three groups, as described in Table 1: "*gripe*" (influenza) word derivations, "*constipação*" (cold) word derivations and flu related symptoms, such as body/throat pains, headache and fever. Using this regular expression, a set of 3183 tweets was identified, an average of 67 tweets per week.

**Table 1:** Regular expressions.

Theme	RegEx
Flu Cold Flu Symptoms	*(en)?grip[a-z]+* *constip[a-z]+* (febre .* grau(s)?) | (grau(s)? .* febre) | (**bodypains **.* febre) | (febre .* **bodypains**)

**bodypains: **do(r(es)?|i-me)\s*(no|na|de|o|a)?\s*(corpo|cabeca|garganta)	

Regular expressions used to filter influenza related tweets.

### Classification methods

Using filtering based on regular expressions as described above is not sufficient, as many tweets that contain words related to flu do not imply that the person writing the text has the flu. Tweets like "*Hoping the flu doesn't strike me again this winter*" contain the keyword flu but do not tell us that this person has the flu. To solve this problem, we applied machine-learning techniques to classify each tweet as "positive" or "negative" according to its content.

#### Manual data annotation

In order to create the predictive models, we asked a group of 37 independent annotators to manually classify a set of tweets, using a simple and intuitive web form. During the annotation task, each annotator was repeatedly assigned a random tweet, with the following restrictions: each tweet had to be labelled by up to three annotators, and each annotator could not label the same tweet more than once. Annotators were instructed to consider a tweet as positive if it revealed that the person who wrote it was with the flu, was having flu symptoms or was recently ill with the flu. A third category was also used, to indicate tweets referring to "cold". We explore the inclusion of these tweets as positive or negative in the results section. To reduce incorrect answers, the annotators could also label the tweet as unknown. Then, a final label was assigned to each tweet according to majority vote, that is, a tweet was considered positive if at least two annotators marked it as positive. Tweets with inconsistent or insufficient labelling information did not receive a final label and were not included in the dataset.

#### Feature extraction and selection

In order to train the classification models, tweets were represented by a bag-of-words (BOW) model. The Natural Language Processing Toolkit [19] (NLTK) was used to tokenize the text, remove Portuguese stopwords and stem all remaining words in each tweet. Character bigrams for each word were also generated, making up a total of 5106 features. Bigrams of words were also tested, but these did not improve the classification results and were therefore removed.

We applied feature selection techniques for defining the best set of features to use. For this, each feature was compared to the true class label to obtain the mutual information (MI) value. The higher a feature's MI score, the more it is related to the true class label, meaning that the feature contains discriminative information to decide if that tweet should be classified as positive or negative. We selected the optimal number of features empirically, by selecting features with MI value above different threshold values and running cross-validation with the training data.

#### Machine learning methods

Several machine learning techniques (SVM, Naïve Bayes, Random Forest, Decision Tree, Nearest Neighbour) were tested in order to evaluate which would produce better results. We used the SVM-light [20] implementation of SVMs. The remaining classifiers were trained using the Scikit-learn toolkit [21].

#### Linear regression models

We used linear regression models to estimate the flu incidence rate, using the Influenzanet data to train and validate the regression. We trained both single and multiple linear regressions, combining the predicted values obtained from the different classifiers, query logs and regular expressions:

(1) $$y_{i}=b_{0}+\overset{K}{\underset{k=1}{\sum }}b_{k}x_{i},k$$

where *y_i _*represents the flu rate in week *i, b*_0 _is the intercept, *x_i,k _*is the value of the predictor *k *in week *i, b_k _*is the coefficient of predictor *k*, and *K *is the total number of predictors used. As the input to the regressions, we used the weekly relative frequencies obtained after applying the regular expressions to the web queries and to tweets, and after classifying the tweets with the various classifiers tested. Also, instead of using the number of positive predictions from the classifiers to calculate the weekly relative frequencies, we tested summing over the classification probabilities of the positive predicted documents in each week, similarly to what was proposed by Culotta [12,13]:

(2) $$f_{i}=\frac{\sum_{d_{j}\in D_{i}}p\left(y_{j}=1|x_{j}\right)}{\left|D_{i}\right|},\;p\left(y_{j}=1|x_{j}\right)>t$$

where *f_i _*is the predicted incidence in week *i, D_i _*is the set of documents for week *i, p*(*y_j _*= 1*|x_j_*) is the probability for classifying document *x_j _*as positive, *|D_i_| *is the number of documents in week *i*, and *t *is the classification threshold.

## Results and discussion

### Data annotation

A total of 7132 annotations were obtained, resulting in 2704 labelled tweets of which 949 were positive for flu. Although a large number of annotators was recruited, which could introduce inconsistencies in the data, this was minimized by the fact that a smaller number of annotators actually contributed to the classification of most of the dataset: the three top annotators contributed to the labelling of 56% of the dataset, and the top ten annotators contributed to 90% of the final labels (Table 2). Moreover, to validate the data obtained from the annotators, the majority voted labels of 500 random tweets were verified by one of the authors, leading to an annotator accuracy of 95.2%.

**Table 2:** Annotators contributions.

Annotator Rank	Cumulative Contribution
1	27.48%
2	44.60%
3	56.25%
4 - 10	90.42%
11 - 37	100.00%

Cumulative percentage of contributed annotations per user. The three users with more annotations contributed to labelling 56.25% of the training data. The top 10 users contributed to labelling 90.42% of the data.

### Binary classification of Twitter messages

The performance of the different classifiers was compared through 5 × 2-fold cross validation using the entire dataset of 2704 tweets, covering the period from May 2011 to February 2012. Using the full set of features, the best results were obtained with the SVM classifier, with an F-measure of 0.75. After feature selection, the best overall results were obtained for a set of 650 features, achieving an F-measure of 0.83 with both SVM and Naïve Bayes classifiers (Table 3).

**Table 3:** Binary classification results.

Classifier	F-Measure	Precision	Recall	AUC
Naïve Bayes	0.83	0.78	0.90	0.941
SVM	0.83	0.78	0.88	0.939
Random Forest	0.81	0.76	0.86	0.934
kNN	0.80	0.72	0.92	0.896
Decision Tree	0.75	0.75	0.75	0.780

Classifier results for the reduced set of features (650 features) after feature selection by mutual information

**Figure 1:**
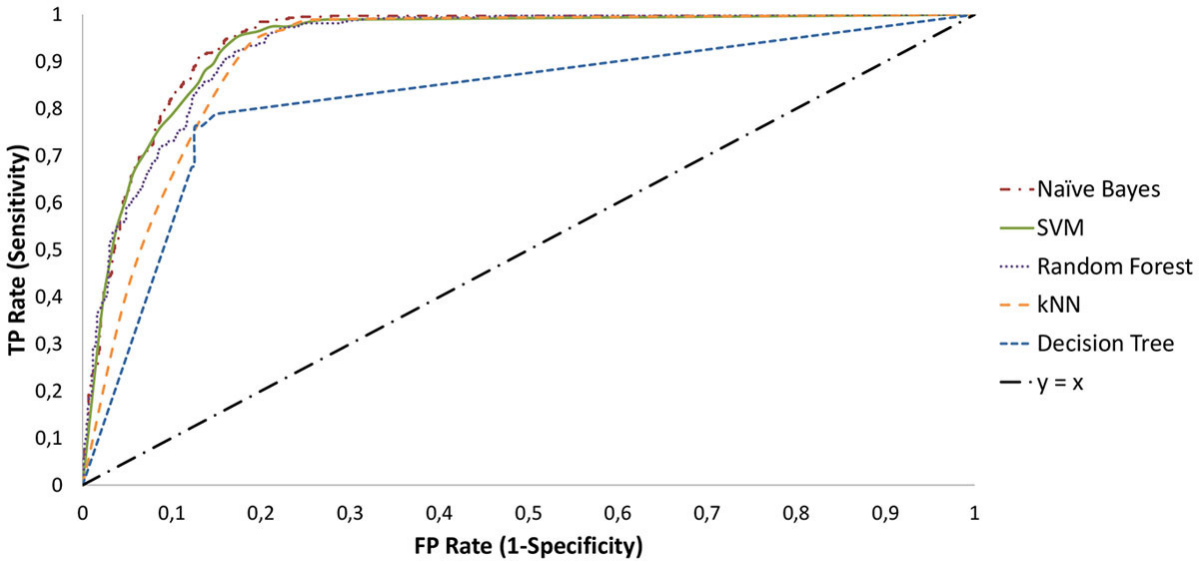
**ROC analysis**. Receiver operating characteristic (ROC) analysis for each classifier, after feature selection.

For each classifier, we selected from the receiver operating characteristic (ROC) analysis (Figure 1) and based on the training data only, an operating point that maximized the classification precision without a severe loss on the classifier recall. Although operating points with higher F-measure values could have been selected, these would represent higher recall, at the expense of a lower precision. We therefore chose the more stringent models, in order to reduce the amount of false positive hits, and consequently, the amount of noise present in the final results.

Applying a simple linear regression between the predictions of each of these classifiers and Influenzanet data resulted in an average correlation ratio of 0.76 for both SVM and Naïve Bayes. When the classifiers were trained with tweets marked as "cold" treated as positive data, the results improved considerably for the Naïve Bayes classifier (0.82) but only a slight change was obtained with the SVM classifier (0.77).

### Flu trend prediction

For flu trend prediction, we tested linear regression models with the relative frequencies calculated from the classification results, query logs and regular expressions as the predictors.

In order to select the best regression model, we executed a series of cross-validation experiments, using data from the period from December 2011 to April 2012 (20 weeks). To avoid overlapping between training and test data, the NB and SVM models for tweet classification were trained with a subset of 1728 manually annotated tweets, covering the period from March 2011 to November 2011.

**Figure 2:**
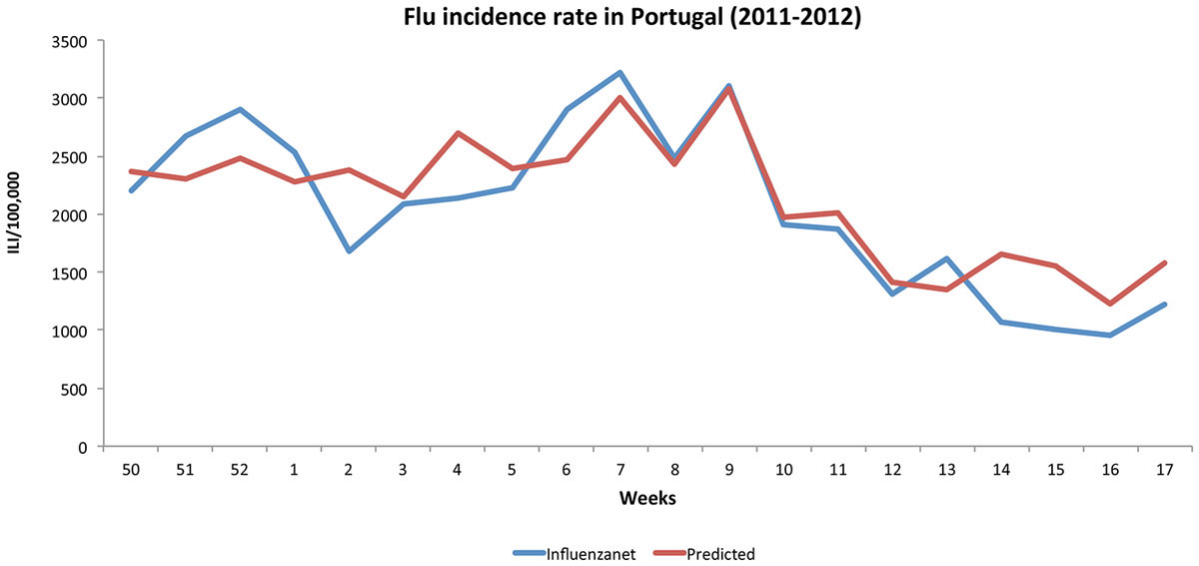
**Multiple linear regression**. Prediction results for the linear regression model using the relative frequencies of queries and of tweets classified by the Naïve Bayes model.

To run the experiments, we randomly partitioned the data into a training set and a test set, each covering ten data points (weeks). This was repeated ten times, and the Pearson's correlation coefficient between the predictor output and the Influenzanet rates, used as gold standard, was calculated for each partition. The average results are shown in Table 4. In the results shown in the table, the Naïve Bayes classifier was used to classify the tweets. A correlation of 0.886 was obtained for a multiple regression combining the queries relative frequency with the tweets relative frequency, using classification probabilities instead of counts in the calculation, as shown in Table 4. For comparison, the best regression obtained with the SVM classifier was achieved with the same configuration, resulting in a correlation ratio of 0.849. Figure 2 shows the resulting predicted trend on this data, using the best regression model. The model was trained with the data for the first ten weeks and applied to the entire time sequence.

**Table 4:** Linear regression results.

		"*cold *" negative	"*cold *" positive
*flu *reg exp		0.837	
*expanded *reg exp		0.801	
Queries		0.571	
Queries + *flu *reg exp		0.849	
Queries + *expanded *reg exp		0.885	

Naïve Bayes	Counts Probabilities	0.761 0.804	0.825 0.824
Naïve Bayes + Queries	Counts Probabilities	0.781 0.794	0.872 **0.886**
Naïve Bayes + *expanded *reg exp	Counts Probabilities	0.770 0.807	0.703 0.791
Naïve Bayes + *expanded *reg exp + Queries	Counts Probabilities	0.873 0.867	0.836 0.801

Pearson's correlation ratios between linear regression estimates and Influenzanet data. *flu *and *expanded *regular expressions correspond to the pattern for "gripe" (flu) word derivations and the complete pattern as shown in Table 1, respectively. The weekly relative frequency was calculated based on the number of positively classified tweets (counts) or on the probabilities given by the classifier (Eq. 2). Tweets referring to "*cold *" were used either as positive or negative data when training the classifier.

One of the possible limitations of this study is the reduced size of the dataset, when compared to similar works. Effectively, despite Twitter being a largely used social web platform, it is not very popular in Portugal, which limited the size of our dataset. As a comparison, we had access to around 14 million tweets as training data, with a daily average of nearly 40,000 tweets, from which 1728 were used to train the binary classifiers. Aramaki et al. [10] used 300 million tweets, from which 5,000 were used for training. On the other hand, Cullota [12] used a total of 500,000 messages, selecting 206 of those messages to train a model. Due to the limited amount of used data, overfitting problems are reported in that work. However, although the data used for calculating the model coefficients was limited, good regression results could still be obtained.

**Figure 3:**
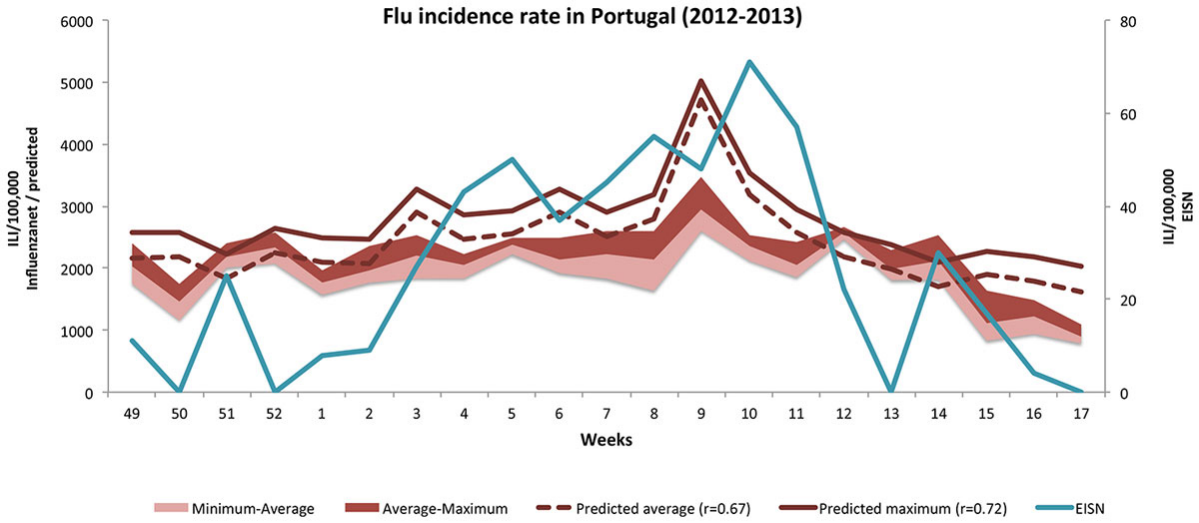
**Flu trend prediction**. Prediction of flu incidence rate in Portugal for the period from December 2012 to April 2013. The models trained with data from the previous flu season were used to generate the prediction. Stacked areas indicate the minimum, average and maximum values registered by the Influenzanet project for each week. The incidence rate reported by the European Influenza Surveillance Network (EISN) is also shown (right axis).

In order to test the hypothesis that the classification and regression models trained in one flu season could be applied in the following season, we trained a NB classification model using the complete set of 2704 annotated tweets and a regression model using the 20 weeks from December 2011 to April 2012. Applying the regular expressions and the previously trained classifier to the 24 million tweets from the period between December 2012 and April 2013, we obtained a total of 5594 positive tweets, representing an average of 266 tweets per week. Similarly, applying the simple regular expression for flu related words to the 14 million queries relative to this period, we obtained 1428 queries, an average of 68 per week. For each week we calculated the relative frequency for queries and tweets, considering classification probabilities as above, and applied the regression model trained with the data from the previous flu season (2011 to 2012). Figure 3 illustrates the regression results obtained.

The Influenzanet project measures the ILI activity levels by week, but updates and reports these results daily taking into account the onset of symptoms in the previous 7 days, and therefore considering weeks starting in each day of the week. Since only the symptom onset is considered, this introduces variability in the data, depending on if we consider the week to start on a Sunday or a Monday. In order to deal with this variability, we considered the average and the maximum reported activity for a given week, for training the regression model. We take Monday as the first day of the week.

The stacked areas in Figure 3 show the minimum, average and maximum ILI activity reported by the Influenzanet project for each week. The solid line shows the predicted trend when the maximum ILI activity value was used to train the regression model. The dashed line shows the trend when the average activity was used. The best result, *r *= 0.72, was obtained considering the maximum activity value in the regression. When the average was considered, the correlation coefficient dropped to *r *= 0.67.

Also shown is the weekly incidence rate as reported by medical doctors and national agencies to the European Influenza Surveillance Network (EISN). The correlation coefficient was in this case much lower, *r *= 0.62, using either the average or the maximum activity values in the regression. However, considering a time delay of one week for this curve in relation to the predicted trend, this correlation increases considerably to *r *= 0.79. In fact, inspecting the lines in the graph, its possible to observe that the EISN trend seems to lag behind the predicted trend by one week. This difference could be a result of the time people take before they visit the doctor, as opposed to the real time nature of social networks.

Another interesting observation from the graph is the seemingly over-estimated peak at week 9 (February 27th to March 4th 2013). In fact, although not indicated by the Influenzanet and EISN values, this corresponds to a period of abnormally high incidence of flu in Portugal, as reported in the media and by the National Institute of Health (INSA) in its weekly flu surveillance bulletin. It is also possible that more references to flu were made in the social networks due to the high impact of news reports. This needs to be inspected in more detail.

## Conclusions

Although many studies targeting the prediction of flu incidence using data from search engine logs or from social media have been presented, this is, to the best of our knowledge, one of the first works on this subject done specifically for the Portuguese language. Although most of the used methods are similar and applicable across languages, the limited amount of available data in languages other than English, as well as language specificities, may influence the final results obtained. Another important novelty of our work is the combination of tweets and user queries, through multiple linear regression models. This contributed to a better approximation to health monitoring results used as gold-standard in this work. A possible extension to this would be to use other sources of user-generated content, such as blog posts and comments on web pages.

The best result reached a Pearson's correlation ratio, between the estimated incidence rate and the Influenzanet data, of 0.89 (*p *< 0.001). This result indicates that this method can be used to complement other measures of disease incidence rates. Unfortunately, the amount of data available for validating the prediction model was reduced, which may limit the relevance of the results.

We also evaluated the application of the classification and regression models from one flu season to the next. The best result, *r *= 0.72, indicates that a good estimate can be obtained, although further work is needed in order to improve this. One possibility to pursue is to apply adaptive learning to update the classification and regression models as new information becomes available, for example from weekly epidemiological reports.

Another important aspect to consider in further studies is whether it is possible to detect in (almost) real-time or predict, with some advance, an increase in the incidence of flu (or other illnesses) in order to optimize the response by the health authorities. The one week delay observed between the EISN data and the predicted trend seems to point in this direction, but this needs further validation.

## Competing interests

The authors declare that they have no competing interests.

## Authors' contributions

JCS performed the data collection and analysis, and helped to draft the manuscript. SM designed and coordinated the study, performed data analysis, and drafted the manuscript. All authors read and approved the final manuscript.

## Declarations

Publication funding for this article has come from FEDER through the Operational Program Competitiveness Factors - COMPETE and from National Funds through FCT - Foundation for Science and Technology, in the context of the projects FCOMP-01-0124-FEDER-022682 (FCT reference PEst-C/EEI/UI0127/2011) and Incentivo/EEI/UI0127/2013.

This article has been published as part of *Theoretical Biology and Medical Modelling *Volume 11 Supplement 1, 2014: Selected articles from the 1st International Work-Conference on Bioinformatics and Biomedical Engineering-IWBBIO 2013. The full contents of the supplement are available online at http://www.tbiomed.com/supplements/11/S1.

## References

[B1] Paul M Dredze M You are what you tweet: Analyzing Twitter for public health Proceedings of the 5th International AAAI Conference on Weblogs and Social Media 2011 265 272

[B2] Scanfeld D Scanfeld V Larson EL Dissemination of health information through social networks: Twitter and antibiotics American Journal of Infection Control 2010 38 3 182 188 10.1016/j.ajic.2009.11.004 20347636 PMC3601456

[B3] Chew C Eysenbach G Pandemics in the Age of Twitter: Content Analysis of Tweets during the 2009 H1N1 Outbreak PLoS ONE 2010 5 11 e14118. 10.1371/journal.pone.0014118 21124761 PMC2993925

[B4] Twitter http://www.twitter.com

[B5] Eysenbach G Infodemiology and infoveillance: framework for an emerging set of public health informatics methods to analyze search, communication and publication behavior on the Internet J Med Internet Res 2009 11 1 e11 10.2196/jmir.1157 19329408 PMC2762766

[B6] Bernardo TM Rajic A Young I Robiadek K Pham MT Funk JA Scoping review on search queries and social media for disease surveillance: a chronology of innovation J Med Internet Res 2013 15 7 e147. 10.2196/jmir.2740 23896182 PMC3785982

[B7] Lyon A Nunn M Grossel G Burgman M Comparison of web-based biosecurity intelligence systems: Biocaster, epispider and healthmap Transboundary and emerging diseases 2012 59 3 223 232 10.1111/j.1865-1682.2011.01258.x 22182229

[B8] Signorini A Segre AM Polgreen PM The use of twitter to track levels of disease activity and public concern in the u.s. during the influenza a h1n1 pandemic PLoS ONE 2011 6 5 e19467 10.1371/journal.pone.0019467 21573238 PMC3087759

[B9] Chunara R Andrews JR Brownstein JS Social and news media enable estimation of epidemiological patterns early in the 2010 haitian cholera outbreak Am J Trop Med Hyg 2012 86 1 39 45 10.4269/ajtmh.2012.11-0597 22232449 PMC3247107

[B10] Aramaki E Maskawa S Morita M Twitter catches the flu: detecting influenza epidemics using Twitter Proceedings of the Conference on Empirical Methods in Natural Language Processing 2011 Association for Computational Linguistics 1568 1576

[B11] Lampos V Cristianini N Tracking the flu pandemic by monitoring the social web Proceedings of the 2nd International Workshop on Cognitive Information Processing (CIP) 2010 411 416

[B12] Culotta A Towards detecting influenza epidemics by analyzing Twitter messages Proceedings of the First Workshop on Social Media Analytics 2010 ACM 115 122

[B13] Culotta A Detecting influenza outbreaks by analyzing Twitter messages 2010 arXiv:1007.4748 [cs.IR]

[B14] Lee K Agrawal A Choudhary A Real-Time Disease Surveillance using Twitter Data: Demonstration on Flu and Cancer Proceedings of the 19th ACM SIGKDD Conference on Knowledge Discovery and Data Mining (KDD): 11-14 August 2013 2013 Chicago, IL. ACM 1474 1477

[B15] Eysenbach G Infodemiology: tracking flu-related searches on the web for syndromic surveillance AMIA Annual Symposium Proceedings: 11-15 November 2006 2006 Washington, DC. AMIA 244 248 PMC1839505 17238340

[B16] Ginsberg J Mohebbi MH Patel RS Brammer L Smolinski MS Brilliant L Detecting influenza epidemics using search engine query data Nature 2009 457 1012 1014 10.1038/nature07634 19020500

[B17] Influenzanet project http://www.influenzanet.eu

[B18] sapo.pt http://www.sapo.pt

[B19] Loper E Bird S Klein E Natural Language Processing with Python 2009 O'Reilly Media Inc

[B20] Joachims T Schölkopf B, Burges C, Smola A Making large Scale SVM Learning Practical Advances in Kernel Methods - Support Vector Learning 1999 MIT-Press

[B21] Pedregosa F Varoquaux G Gramfort A Michel V Thirion B Grisel O Blondel M Prettenhofer P Weiss R Dubourg V Vanderplas J Passos A Cournapeau D Brucher M Perrot M Duchesnay E. Scikit-learn: Machine Learning in Python Journal of Machine Learning Research 2011 12 2825 2830

